# Fourier-Transform InfraRed (FT-IR) spectroscopy to show alterations in molecular composition of EV subpopulations from melanoma cell lines in different malignancy

**DOI:** 10.1016/j.bbrep.2020.100888

**Published:** 2021-01-04

**Authors:** Ewa Ł. Stępień, Agnieszka Kamińska, Magdalena Surman, Dagmara Karbowska, Andrzej Wróbel, Małgorzata Przybyło

**Affiliations:** a Department of Medical Physics, Marian Smoluchowski Institute of Physics, Faculty of Physics, Astronomy and Applied Computer Science, Jagiellonian University, 30-348, Kraków, Poland; b Department of Glycoconjugate Biochemistry, Institute of Zoology and Biomedical Research, Faculty of Biology, Jagiellonian University, 30-387, Kraków, Poland

**Keywords:** Extracellular vesicles, Fourier transform infrared spectroscopy, Melanoma, Protein secondary structure

## Abstract

**Background:**

Melanoma cells release extracellular vesicles (EVs) subpopulations which differ in size, phenotype and molecular content. Melanoma derived EVs play a role in the development and progression of cancer by delivering surface receptors and bioactive (proteins, lipids, nucleic acids) or signaling molecules to target cells.

**Methods:**

We applied Fourier Transform Infrared Spectroscopy (FTIR) to compare infrared spectra of absorption for different subpopulations of EVs originating from normal human melanocytes, primary cutaneous melanoma (WM115) and metastatic cutaneous melanoma (WM266-4).

**Results:**

FTIR results showed that exosome and ectosome populations differ in content of protein and lipid components. We obtained higher lipid to protein ratio for ectosomes in comparison with exosomes what confirms that exosomes are very densely packed with protein cargo. We identified the lowest value of saturated fatty acids/unsaturated fatty acids parameter in the metastatic WM266-4 cell line and ectosomes derived from WM266-4 cell line in comparison with normal melanocytes and the primary WM115 cell line. We identified the alterations in the content of secondary structures of proteins present in EV subpopulations originating from melanocytes and melanoma cells in different malignancy.

**Conclusions:**

Obtained results revealed differences in the molecular composition of melanoma derived EVs subtypes, including protein secondary structure, and showed progressive structural changes during cancer development.

## Highlights

• Fourier-Transformed Infrared spectroscopy allows recognition lipid and protein content in extracellular vesicles (EVs).
• Subpopulations of (EVs) from human melanocytes and melanoma cells contain distinct lipid composition and protein structure.
• Ectosomes from malignant human melanoma are rich in saturated fatty acids and random coiled proteins.
• Exosomes from malignant human melanoma are bigger in compare to those from melanocytes and have higher lipid to amid ratio.

## Graphical abstract

**Figure:**
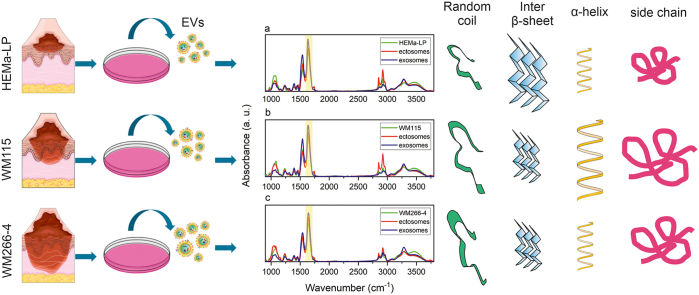

## 1 Introduction

Malignant melanoma is one of the most aggressive and the deadliest type of skin cancer which occasionally develops in other organs (eye, oral and nasal mucosa, vulval and anorectal mucosa) [1]. The biggest challenge of contemporary melanoma diagnostics is early identification of this cancer to offer the successful treatment for a patient [2]. During melanoma development, its metabolism is reprogrammed what contributes to the pathogenesis and heterogeneity of melanoma phenotype like pigmentation (melanin granule content) or protein glycosylation profile (complex-type glycans) [[3], [4], [5], [6]]. This melanoma development towards more malignant forms results in changes in their biological characteristics (migration, proliferation), genetic profile, as well as physical properties (cell elasticity, membrane hydration level) [3,[7], [8], [9]].

Melanoma cells release a variety of extracellular vesicle (EV) subtypes including smaller exosomes (<150 nm) and larger ectosomes (>100 nm) diverse phenotype and molecular content changing within melanoma treatment [10]. Melanoma EVs play a role in the development and progression of cancer by delivering surface receptors, bioactive (proteins, lipids, nucleic acids) or signaling molecules to target cells [11,12]. EVs may be utilized as a source of prognostic and diagnostic biomarkers, and the finding the disease resulting changes in EV proteome makes an additional impact in cancer biomarkers investigation [10,13]. Previously, the alterations in protein content of EVs produced by different glioblastoma subtypes have been demonstrated, what confirmed that EVs can be used as indicators of glioblastoma (GBM) aggressiveness and assist in GBM classification [14]. Changes in melanoma chondroitin sulfate proteoglycan (MCSP), melanoma cell adhesion molecule (MCAM) and other proteins were documented in EVs from patient-derived melanoma cell lines and conformed in melanoma patients receiving targeted therapy [7,10,13].

Previous reports led us to set up a goal to find more comprehensive methodology for recognition differences in melanoma EVs molecular content. We aimed to conform Attenuated Total Reflection Fourier Transform Infrared (ATR-FTIR) spectroscopy to obtain infrared spectra of absorption for different subpopulations of EVs. The main objective of this study was to find differences in the ATR-FTIR spectral signatures for exosomes and ectosomes which were representative for the human melanoma cell lines showing different levels of metastasis (primary and malignant melanoma cell line) in comparison with normal human melanocytes.

## 2 Materials and methods

### 2.1 Cell lines and cell culture conditions

Three different cell lines: human epidermal melanocytes (HEMa-LP), primary cutaneous melanoma (WM115) and metastatic cutaneous melanoma (WM266-4) were used for the study. HEMa-LP (cat. No. C0245C, Thermo Fisher Scientific, MA, USA) were cultured on T75cm^2^ cell culture flask in M254 medium (cat. No. M-254-500, Gibco™ Paisley, UK) supplemented with Human Melanocyte Growth Supplement-2 (cat. No. S-002-5, Gibco™ Paisley, UK) and antibiotics: 100 U/mL penicillin and 100 μg/mL streptomycin (cat. No. 15140122, Gibco™ Paisley, UK).

Two melanoma cell lines were obtained from the ESTDAB Melanoma Cell Bank (Tübingen, Germany). WM115 (ESTDAB-066, primary cutaneous melanoma) and WM266-4 (ESTDAB-076, metastatic cutaneous melanoma) originated from the same individual and represented radial/vertical growth phase and lymph node metastasis, respectively [15]. Melanoma cells were maintained on 100 mm dishes in RPMI 1640 medium with GlutaMax-I (cat. No. 72400112, Thermo Fisher Scientific, MA, USA), supplemented with 10% FBS (cat. No. 10270106, Thermo Fisher Scientific), penicillin (100 U/mL) and streptomycin (100 μg/mL) (cat. No. P4333, Sigma-Aldrich, St. Louis, MI, USA).

Cells were grown in monolayers in 5% CO_2_ atmosphere at 37 °C in a humidified incubator and passaged after reaching 80% confluence.

### 2.2 Separation of EVs subpopulations from melanocytes and melanoma cell lines

**Fig. 1:**
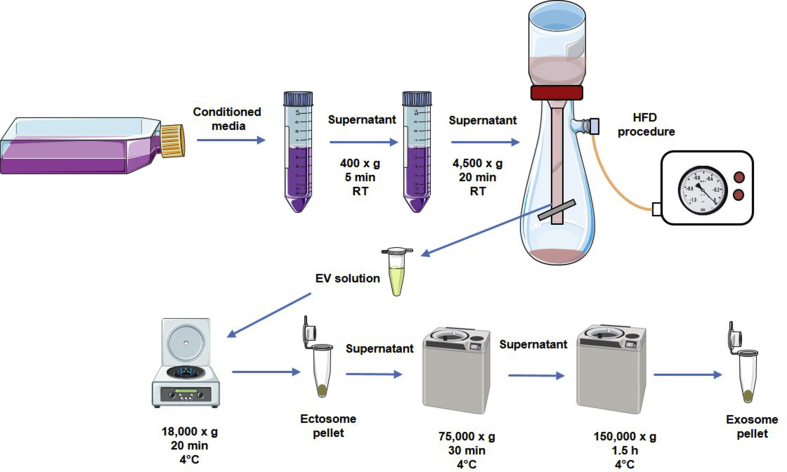
Workflow scheme for EV separation to obtain ectosomes and exosomes from cell culture media.

Two subpopulations of EVs: exosomes and ectosomes were separated from 80 mL of cell culture media collected from each cell line. Before this procedure, sub-confluent cells were cultured 24 h in serum free medium supplemented with antibiotics. Collected media were centrifuged subsequently at 400×*g* (5 min, 4 °C) and 4500×*g* (20 min, 4 °C) to remove remaining cells and cellular debris. Then, supernatants were used for hydrostatic filtration dialysis (HFD) method assisted by a low vacuum pump (0.2 mBar). For EVs concentration, dialysis membrane with 1000 kDa molecular weight cut-off (MWCO) was applied [16]. The retained EV solution (1 mL) was divided into two equal parts. Firstly, EV solutions were centrifuged in an Eppendorf 5424 R at 18 000×*g* for 20 min at 4 °C to obtain ectosomes pellet. Then, the supernatant was collected and ultracentrifuged (Sorvall MX 150+ Micro-Ultracentrifuge, Thermo Fisher Scientific, MA, USA) using S140-AT fixed angle rotor (cat. No. 45978, Thermo Fisher Scientific, MA, USA) at 75 000×*g* for 30 min at 4 °C and followed by centrifugation at 150 000×*g* for 1.5 h to get exosomes (Fig. 1). In each case, one of the parts was used to prepare the sample for TEM visualization and another one was suspended in 50 μl of deionized water and used for other measurements.

### 2.3 EV size distribution and concentration analysis by Nanoparticle Tracking Analysis

The size distribution and concentration of isolated EV subpopulations were determined by Nanoparticle Tracking Analysis (NTA) method using NanoSight LM10 Malvern system (Malvern Panalytical Ltd, UK) equipped with a sCMOS camera and a 405 nm laser. Before measurement, the exosomes and ectosomes samples were diluted in a filtered (0.05 μm) deionized water. The assay was performed at room temperature (22 °C) and 5 videos for 30s were recorded for EV samples. Data were captured and analyzed using NTA 3.1 Build 3.1.45 software with a detect threshold of 5 and camera level of 11.

### 2.4 EV visualization by Transmission Electron Microscopy

For preparation of Transmission Electron Microscopy (TEM) images, exosomes and ectosomes pellet from each cell line was fixed with 2.5% GA (cat. no. G5882, Sigma-Aldrich, St. Louis, USA) in 0.1 M cacodylic buffer (cat. no. C4945, Aldrich, St. Louis, USA) for 2 h at RT. Then, samples were postfixed in 1% osmium tetroxide solution (1 h) and dehydrated by passing through graded ethanol series and embedded in PolyBed 812 at 68 °C. Ultrathin sections were collected on 300 mesh grids or one slot made from copper, additionally the latter was covered with formvar film. For cutting the Leica EM UC7 microtome was used. Then, sections were contrasted using uranyl acetate and lead citrate. For observation, the JEOL JEM 2100 H T electron microscope (Jeol Ltd, Tokyo, Japan) was used at accelerating voltage 80 kV.

### 2.5 Attenuated Total Reflection Fourier Transform Infrared (ATR-FTIR) spectroscopy measurements

ATR-FTIR spectra were collected using a Nicolet 6700 (Thermo Fisher Scientific, MA, USA) spectrometer equipped with diamond ATR accessory. 5 μl of sample (exosomes, ectosomes or cell line) was mounted on the ATR crystal and left to dry. The measurements were performed at room temperature and 256 scans were co-added at a nominal resolution of 4 cm^−1^. Collected spectra were postprocessed by smoothing (Savitzky-Golay filter of polynomial order 2 to data frames of length 7), background substracting and normalization using SpectraGryph v. 1.2.13 software [17]. All spectra were normalized to the value of the area under the spectra equal to 1. Second derivative calculation, band fitting and statistical calculations were performed in OriginPro 2019b (Northampton, MA, USA). Graphs were plotted using Grapher 11 (Golden Software, USA). Based on previous measurements of FTIR spectra for the same samples (HEMa-LP, WM115, WM266-4 cell lines and EV subpopulations derived from these cell lines), we calculated the standard deviations (SD) and means for each wavenumber of spectra (Fig. 4). The value of SD is a precision of measurement. We applied the similar methodology of assuming a precision of following spectral parameters: SFA/UFA, ACL and Lipid/Amid ratios. Firstly, we calculated above mentioned parameters for each spectra measured previously. We calculated standard deviation, mean and coefficient of variation (COV) for each parameter. We assumed that COV obtained for earlier measurements have the same value for spectra in the present study. Finally, we calculated the coefficient of variation (COV). We assumed that COV obtained for earlier measurements have the same value for spectra in the present study.

Based on the COV value, we estimated the precision of spectral parameters (Fig. 6, Fig. 7).

### 2.6 Analysis of EV protein secondary structure

The secondary structure of proteins present in EVs was determined by decomposing the second derivative of the Amid I band using Peak Deconvolution App available in OriginPro 2019b (Fig. 5c). Amid I (1600–1700 cm^−1^) which corresponds to C=O (70–85%) and C–N (10–20%) vibrations, is the most sensitive spectral region to protein secondary structure compositions [18]. All second derivatives were multiplied by −1 and baseline corrected as was presented by Usoltsev D et al. [19]. We calculated standard errors of parameters determined during the process of peak fitting using OriginPro 2019b (Fig. 7).

## 3 Results and discussion

### 3.1 Characterization of EV size distribution by NTA

**Fig. 2:**
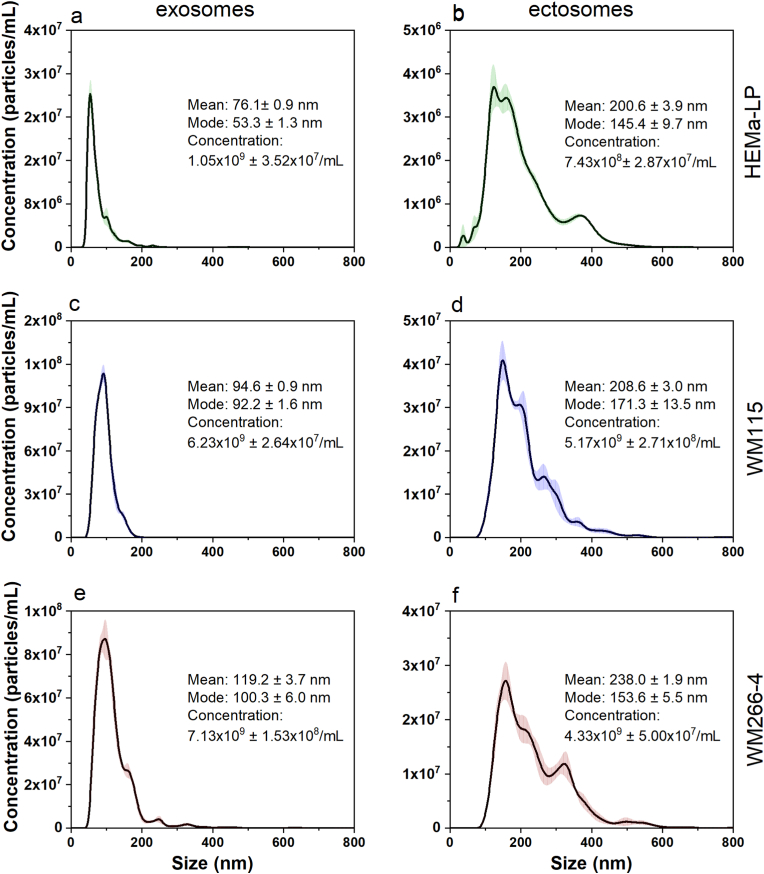
The size distribution of EV subpopulations obtained by NTA method: exosomes and ectosomes from HEMa-LP (a, b), WM115 (c, d) and WM266-4 cell line (e, f). The size distributions are depicted as mean (solid line) with standard error (transparent area). The concentration was calculated taking into account the dilution factor of the samples.

Size distribution of EV subpopulations corresponds to the hydrodynamic diameters of the EVs in suspension. Both mean and mode of the hydrodynamic diameter were lower for exosomes in comparison with ectosomes (Fig. 2). The exosome size was in the range of 30–200 nm, while the ectosomes subpopulation was in the rage of 50–600 nm, which proves that these subpopulations were properly separated. Interestingly, we observed a shift towards bigger exosome mean size for exosomes obtained from more malignant cells, the smallest exosomes were obtained from melanocytes (HEMa-LP), the biggest were derived from the most malignant cell line (WM266-4).

### 3.2 Visualization of EV subpopulations by TEM

**Fig. 3:**
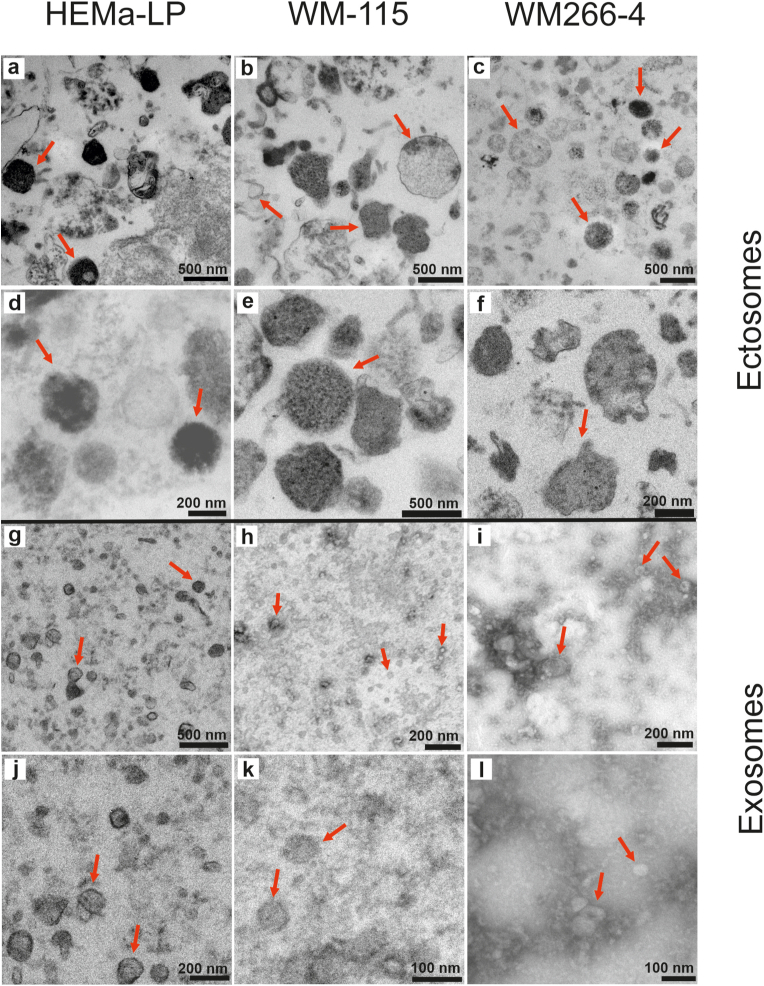
Representative TEM images of EV subpopulations from each studied cell line. EV subtypes have been shown at three different magnification: ectosomes (a–f) and exosomes (g–l). Ectosomes reaches size about 500–600 nm. In contrast, exosomes reach size up to 200 nm. EVs were marked with red arrows. (For interpretation of the references to colour in this figure legend, the reader is referred to the Web version of this article.)

TEM visualization was performed for screening the morphology and size of EV subpopulations originating from studied cell lines. The representative TEM images of EV derived from melanocytes and two melanoma cell lines: WM115 and WM266-4 are shown in Fig. 3. Obtained images confirmed the lipid membrane integrity of EV subtypes after purification procedures and separation between smaller in size exosomes (Fig. 3g-l) and larger ectosomes (Fig. 3a–f). For each type of vesicles, we observed diversity in terms of electron density, which indicated a different molecular content within both exosome and ectosome populations. Especially for HEMa-LP ectosomes, we observed more dense “black” granules (Fig. 3a, d) having probably more melanin. TEM images revealed that exosomes derived from WM115 and WM266-4 cell lines have a tendency to aggregate compared to exosomes originating from melanocytes (Fig. 3k and l). These results supported NTA data (Fig. 2c, e).

### 3.3 General molecular composition of melanocytes and melanoma cell lines and their respective EV subpopulations

**Fig. 4:**
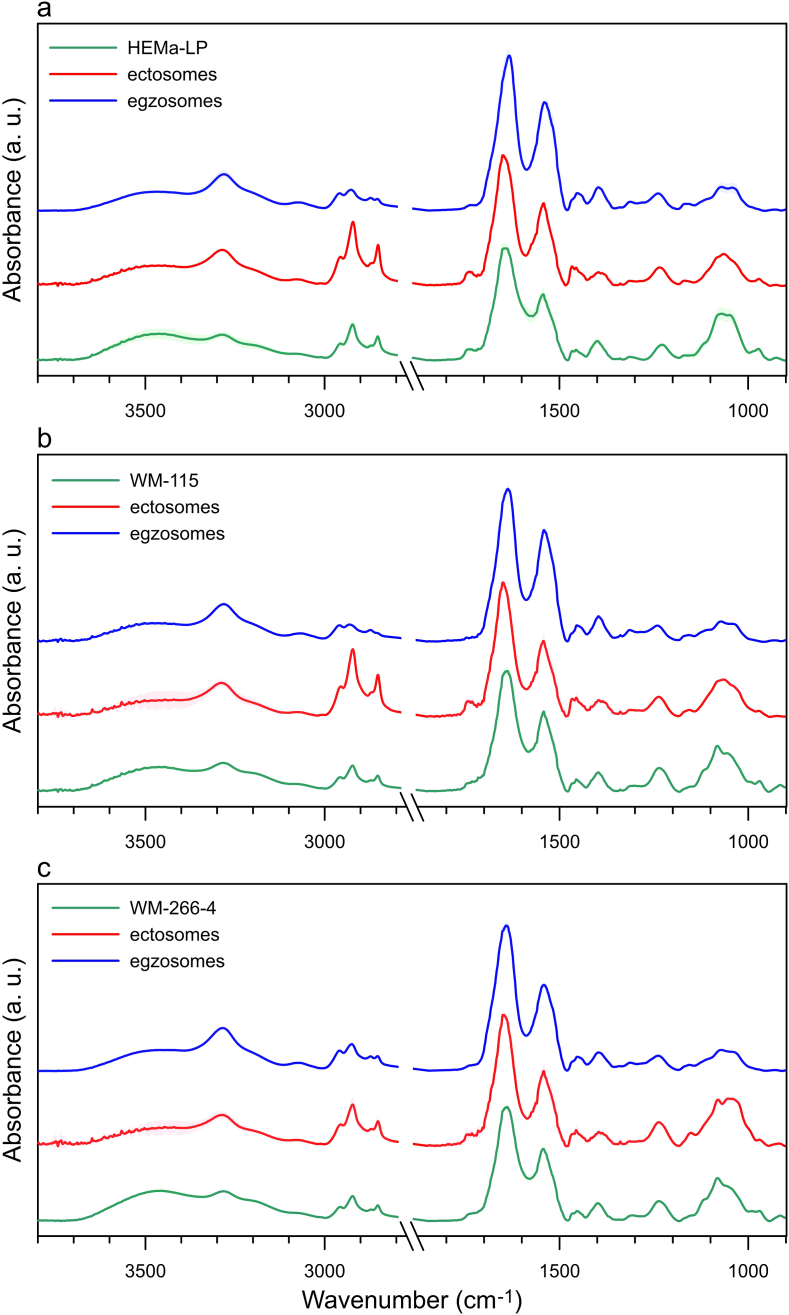
The average ATR-FTIR spectra of cells, exosomes and ectosomes originated from HEMa-LP (a), WM115 (b) and WM266-4 cell line (c), respectively.

To characterize molecular composition of human melanocytes, two melanoma cell lines, and ectosome and exosome subpopulations isolated from these cell lines, the ATR-FTIR spectra were gathered and analyzed. ATR-FTIR spectrum in the mid-infrared range consists primarily of lipid and amid components (Fig. 4a). In the amide component we can distinguish Amid I (1650 cm^−1^, originating mainly from C=O vibrations of the protein peptide backbone) and Amid II (1540 cm^−1^, originating from N–H vibrations of the peptide groups) bands [20]. The average ATR-FTIR spectra obtained for studied cell lines and EV subpopulations (exosomes and ectosomes) were presented in Fig. 4a, b, c. Also, the changes of the protein absorption region of the acyl chain stretching modes were observed, showing potential protein modifications in the different EV subpopulations [21].

The lipid composition of EVs is typical as for the cell plasma membrane but the appearance of their spectra differs from the typical distribution to some extend because they are affected by EV biogenesis process. In our study, the relative absorbance of lipid bands (2800–3000, 1734 cm^−1^) was much higher for ectosomes in comparison with exosomes, which means that membrane components are very representative for ectosomes. Ectosomes are formed from the cell membrane by blebbing and they are enriched in phosphatidylserine (PS), sphingomyelin (SM), cholesterol or diacylglycerol, which are present in both inner and outer leaflet of cell membrane [7,22], while the main lipid component of exosomes is cholesterol [23].

For the measured exosome samples, we found a higher absorbance for characteristic bands of proteins (3284, 1647, 1545 cm^−1^), which means that exosomes are very densely packed with protein cargo. Our observations are in line with results obtained by Mihaly et al. who showed the higher protein to lipid ratio for exosomes compared to ectosomes isolated from Jurkat cell line [20]. In ATR-FTIR spectrum we can also observe characteristic bands for: Amid A (3290 cm^−1^), Amid B (3078 cm^−1^) and DNA (970 cm^−1^) [20,24,25].

### 3.4 Lipid composition of melanocytes and melanoma cell lines and their respective EV subpopulations

**Fig. 5:**
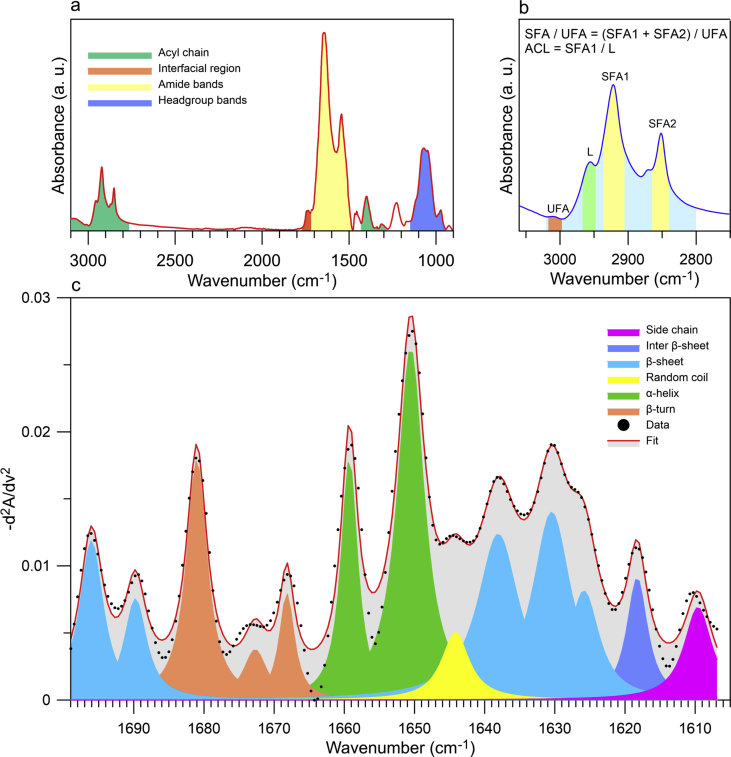
Components of the ATR-FTIR spectrum characteristic for amide bands and components of lipids: acyl chain, interfacial region and headgroup (a) and method of calculating saturated to unsaturated fatty acid ratio (SFA/USA) (b). Second derivative deconvolution of the Amide I band frequencies and assignments to secondary structure for proteins present in the measured samples (c). The assignment of wavenumber to particular secondary structure of protein was made on the basis of literature [8,9,29].

The bands characteristic for acyl chain (3000–2800, 1396 cm^−1^), headgroup (1300-1000 cm^−1^) and interfacial region (1743, 1728 cm^−1^) can be specified in the lipid component present in ATR-FTIR spectrum [[26], [27], [28]]. Moreover, in the lipid component we can distinguish bands characteristic for saturated (2922, 2853 cm^−1^) and unsaturated (3006 cm^−1^) fatty acids what has been shown in Fig. 5b [29]. Based on the ATR-FTIR spectra, according to Dogan A et al. [28], selected parameters have been calculated to characterize more thoroughly lipid composition of EVs: the saturated to unsaturated fatty acids (FAs) ratio (SFA/UFA), acyl chain length (ACL) and the Lipid to Protein ratio for paternal cells and their derivative EVs (Fig. 5a). To calculate above mentioned parameter the following spectral ranges were used: SFA1: 2905-2936 cm^−1^, SFA2: 2840-2861 cm^−1^, UFA: 2977-3017 cm^−1^, L: 2950-2966 cm^−1^ (see Fig. 5b).

The secondary structure of proteins presents in cell lines and EVs was determined by decomposing the second derivative of the Amid I [30,31]. The detailed assignment of ATR-FTIR wavenumber to a given secondary structure of protein is presented in Fig. 5c. The process of peak fitting with calculated parameters has been presented in detail in the Supplementary material file (Fig. S1) and numeric fitting data are in the Fit_Results.xlsx file.

It has been previously reported that lipid composition of cancer cells varies from normal cells due to different lipid metabolism and synthesis under stressing conditions [32]. Such differences in the levels of the membrane lipids, such as PCs, SMs, FAs, PEs, and phosphatidylinositols (PIs) were observed in seven breast cell lines, and the degree of FA unsaturation (number of double bounds C=C) correlated with cancer stage and malignancy [33]. However, data appear to be largely inconsistent and still it is scarcely known about lipid metabolism in melanoma metastasis [34].

**Fig. 6:**
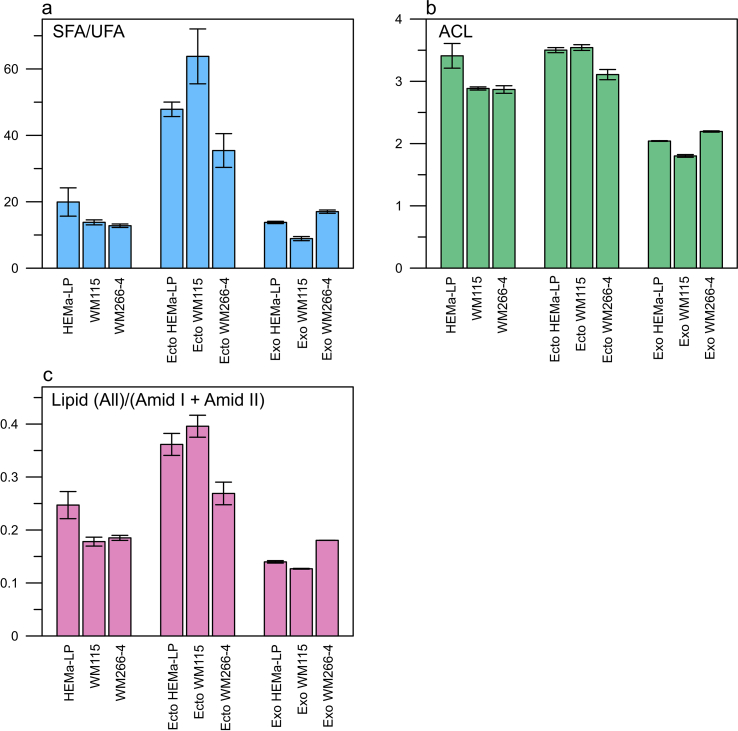
Relative values of selected IR spectral parameters: the SFA/UFA ratio (a), acyl chain length (b) and the Lipid/Protein ratio (c) obtained for cell lines (HEMa-LP. WM115, WM266-4) and EVs (ectosomes, exosomes) derived from each cell line. These parameters were used for the quantitative evaluation of compositional differences in measured cell lines and their respective EV subpopulations.

In our study we observed that the SFA/UFA ratio is lower in ectosomes derived from the metastatic WM266-4 cell line in comparison with normal melanocytes and the primary WM115 cell line (Fig. 6a). Interestingly, ectosomes had much more SFA then whole cells and exosomes.

Not only lipid composition is different in ectosomes but also the Lipid/Protein ratio is much higher as compared with representative exosomes and cells (Fig. 6c).

Phosphatidylcholines (PCs) are the major components of membrane lipids and their contribute to proliferative growth in cancer cells since the synthesis was increased in response to *de novo* FAs synthesis [35]. Our data show a higher absorbance for characteristic band of PCs and PEs (1062 cm^−1^) in WM266-4 ectosomes in comparison with those derived from WM115 or normal melanocytes (Fig. 4, Fig. 5a).

In addition to changes in the degree of saturation of lipid chains, the changes in the length of acyl chains are also observed. Naguib et al. showed that length of acyl chains is reduced in PIs as a result of mutation of p53 protein which is common in cancers including melanoma [36,37]. We observed reduced ACL parameter in WM115 and WM266-4 cell lines compared to melanocytes (Fig. 6b). In case of EV subpopulations, the value of ACL was lower in ectosomes from WM266-4 compared to other cell lines.

Protein-to-lipid ratio parameter that enable discrimination of different EV subpopulations was for the first time proposed by Osteikoetxea et al. [38] It has been shown that the value of protein/lipid parameter is higher in exosomes compared to ectosomes subpopulation what confirms the existence of the sorting mechanism during the generation of different EV subpopulations [39]. These results correspond with our observations.

### 3.5 Variations in the percentage content of given protein secondary structures

**Fig. 7:**
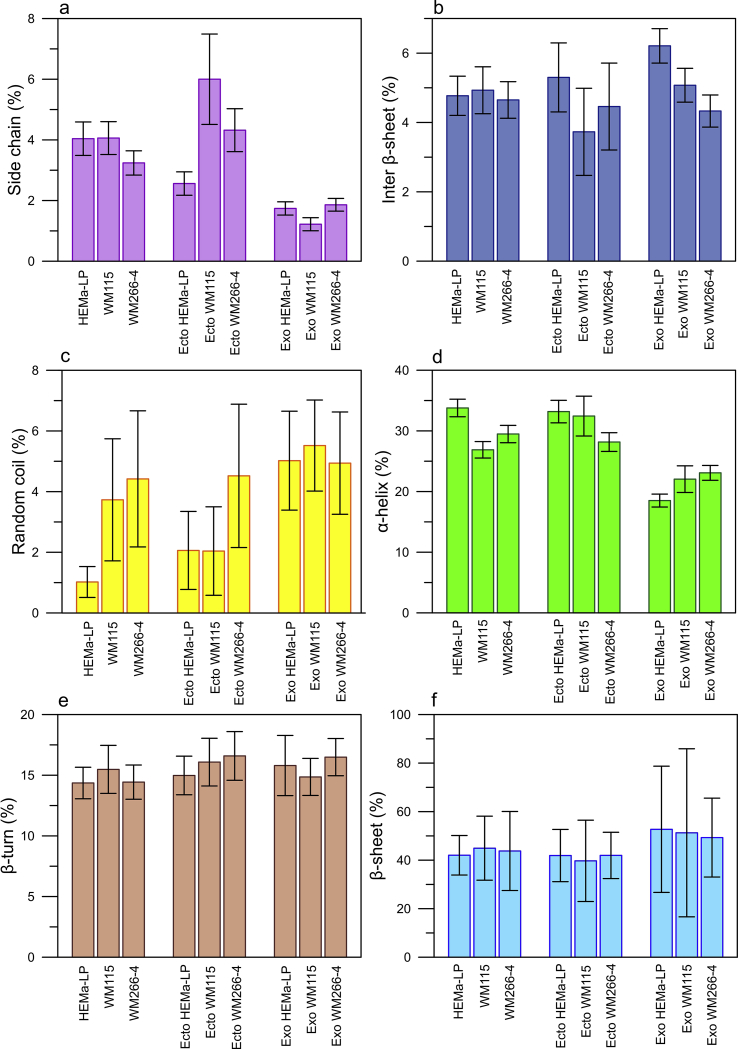
The percentage of given protein secondary structures (±the standard errors of area under the fitted peak) in measured cell lines (HEMa-LP, WM115, WM266-4) and respective EV samples (ectosomes, exosomes).

In the present study an increase abundance in intermolecular β-sheet structure was observed in melanocyte-derived ectosomes and exosomes compared to EVs originating from melanoma cell lines, while the percentage content of β-sheet structure was almost the same in cells from studied cell lines (Fig. 7b). We also observed a gradual decrease in the content of inter-β and β-sheet structure in exosomes from melanocytes to the most malignant form of melanoma (WM266-4) (Fig. 7b, f). On the contrary, the increasing content of α-helix structure in exosomes correlated positively with the degree of malignancy (Fig. 7d), while the cellular α-helix content decreased. Similarly, in other studies decreased content of α-helical structures distinguished malignant melanoma biopsies from benign pigmented nevi, basal cell carcinoma and seborrheic keratosis [40]. Such intensified release of proteins enriched in α-helix structure in EVs from more malignant melanoma cells might be a potential mechanism that allows cancer cells to gain their metastatic potential.

The most significant differences in the present study concerned the content of random coil structure between melanocytes and WM266-4 cells (Fig. 7c). The highest percentage of random coil structure was found in WM266-4 cells, in ectosomes from WM266-4 and exosomes from WM115 cell line. Our observations concerning EV proteins are consistent with those of Anastassopoulou et al. who demonstrated that the secondary structure of cellular proteins changes from α-helix to β-sheet and random coil during melanoma development and progression [41]. The finding that ATR-FTIR can detect the shift towards random coil structure in cancer cells is furtherly supported by studies involving two bladder cancer lines (T24 and UC13), where increased random coil/α-helix ratios were found [42]. Similarly, disordered protein conformation were more predominant in colonic adenocarcinoma, in comparison to normal colonic mucosa [43].

This higher abundance of intrinsically disordered proteins (IDPs) is strongly associated with cancer. IDPs range from fully unstructured to partially structured and include random coils. They are significantly depleted in order-promoting hydrophobic (Ile, Leu, and Val) and aromatic (Trp, Tyr, and Phe) amino acid residues, and possess a low content of Cys and Asn residues. On the other hand, IDPs are enriched in disorder-promoting polar (Arg, Gly, Gln, Ser, Pro, Glu, and Lys) and structure-breaking (Gly and Pro) amino acids [44]. In a study by Iakoucheva et al. [45] 79% of cancer-associated proteins were found to contain predicted regions of disorder that were 30 residues or longer. In contrast, only 13% of proteins from a set of proteins with well-defined ordered structures contained such long regions of predicted disorder. In experimental studies the presence of disorder has been directly observed in several cancer-associated proteins, including proto-oncogene p53, alpha-fetoprotein (AFP), and breast cancer type 1 susceptibility protein (BRCA1) [42].

Regarding melanoma, typical mutations are observed in genes coding proteins such as B-Raf serine/threonine kinase (BRAF), cyclin-dependent kinase 4 (CDK4), melanocyte inducing transcription factor (MITF), phosphatidylinositol 3,4,5-trisphosphate 3-phosphatase (PTEN), cyclin-dependent kinase inhibitor 2A (CDKN2A), ARF tumor suppressor (p14ARF), and p53. These proteins are present in a few functional proteoforms with disordered structures, adding another route to the disease's etiology [46]. In addition, the oncogenic form of GTPase KRas (KRAS) was identified as an intrinsically disordered protein [47], and was shown to drive cancer progression in mouse model of melanoma [48].

Finally, following changes in protein secondary structure with ATR-FTIR may also be useful to classify blood sera from breast cancer and healthy donors. However, the protein composition (total secondary structure of the protein mixture) cannot contribute to distinguish serum samples from these groups of patients, because the contribution of protein structure in a total serum protein mixture is too small [49]. Using EVs as protein carriers to characterize the α-helix and β-sheets will target better metabolic differences between normal and cancer cells.

## 4 Conclusions

The main goal of the present study was to find differences in the ATR-FTIR spectral signatures for EV subtypes (exosomes and ectosomes) derived from melanoma cell lines showing dissimilar levels of metastasis (WM115 and WM266-4) compared to normal human melanocytes (HEMa-LP).

Based on the registered ATR-FTIR spectra for cell lines and EV subtypes derived from parental cells, we calculated parameters that enable differentiation of studied samples such as: Lipid (All)/(Amid I + Amid II), ACL and SFA/UFA. Our experiment confirmed previous observations that exosome and ectosome populations differ in content of protein and lipid components. We obtained higher lipid to protein ratio for ectosomes in comparison with exosomes what confirms that exosomes are very densely packed with protein cargo.

Analysis of the lipid component in the ATR-FTIR spectra allowed to find differences in the content of saturated and unsaturated fatty acids (SFA/UFA ratio) in studied samples. We identified the lowest value of SFA/UFA parameter in the metastatic WM266-4 cell line and ectosomes derived from this cell line in comparison with normal melanocytes and the primary WM115 cell line. These results suggest that lipid composition of WM266-4 derived ectosomes can be potential prognostic biomarker for metastasis. Interestingly, we also found reduced value of ACL parameter in WM115 and WM266-4 cell lines compared to melanocytes. The same relationship was observed in ectosomes from WM266-4 cell line. The findings of this study suggest that exosomes are promising source of protein biomarkers for melanoma metastasis, while ectosomes are potential source of lipid biomarkers.

One of the more significant findings to emerge from this study is that FTIR analysis allows to identify the alterations in the content of secondary structures of proteins present in EV subpopulations originating from melanocytes and melanoma cells in different malignancy. We found differences in the percentage content of random coil, β-sheet, inter β-sheet and α-helix between studied samples. The most significant differences in the present study concerned the content of random coil structure between melanocytes and WM266-4 cells. The highest percentage of random coil structure was found in WM266-4 cells, in ectosomes from WM266-4 and exosomes from WM115 cell line.

The results of this study indicate that the molecular composition of EVs subtypes, including protein secondary structure is changing during melanoma development. These molecular changes can be quickly identified by FTIR analysis and then used as a marker for metastasis development.

## Funding

This work was supported by grants from the Jagiellonian University (N17/MNS/000002 106) and from the Polish National Science Centre (2018/31/N/NZ4/03787).

## Declaration of competing interest

The authors declare that they have no conflicts of interest.
